# Modified Fasanella-Servat for Acquired Ptosis: Case Report and Review of the Literature

**DOI:** 10.4103/0974-9233.58415

**Published:** 2009

**Authors:** Imtiaz A Chaudhry

**Affiliations:** From the Oculoplastic and Orbit Division, King Khaled Eye Specialist Hospital, Riyadh, KSA

**Keywords:** Acquired Ptosis, Complications, Fasanella-Servat, Surgery

## Abstract

A 28-year-old man who had acquired ptosis of his left upper eyelid after a traffic accident did not benefit from standard levator advancement surgery. Patient had significant ptosis with moderate levator function. A modified Fasanella-Servat procedure under local anesthesia resulted in the desired correction of his left upper eyelid ptosis. A review of the Fasanella-Servat procedure for ptosis surgery is presented, as well as its modifications, along with its limitations.

## CASE REPORT

**Figure 1:**
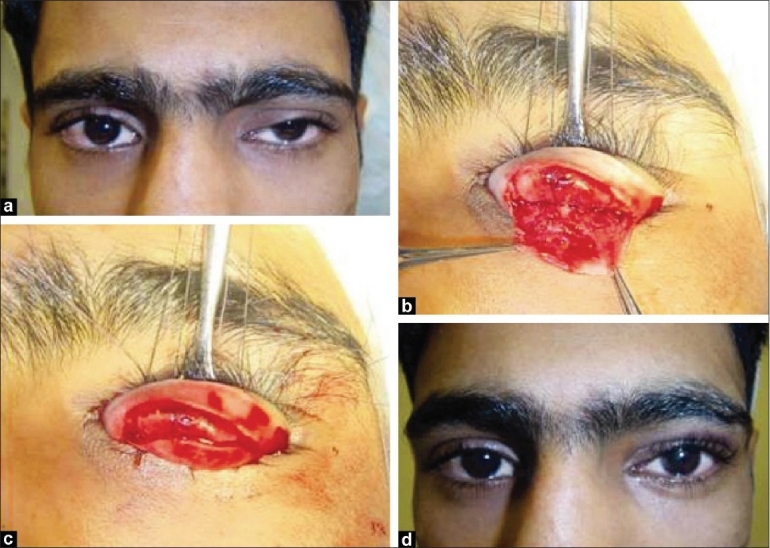
(a) Patient with significant ptosis and moderate levator function before modified Fasanella-Servat procedure; (b and c) Modification of Fasanella-Servat procedure: After eyelid eversion, incision through tarsus approximately 3 mm below superior tarsal border is made, and dissection between Mueller's muscle and levator aponeurosisis carried upward. Sutures are passed through superior tarsus, Mueller's muscle and conjunctiva, superior edge of the tarsus and anterior lamella of the eyelid to emerge on the skin surface, where they are tied; (d) Patient after undergoing Fasanella-Servat procedure for correction of left upper eyelid acquired ptosis, demonstrating good results

A 28-year-old man was referred for evaluation of posttraumatic ptosis of his left upper eyelid after an attempt to repair the acquired ptosis had failed. His past medical and ocular history, as well as family history, was noncontributory. Patient had sustained road-traffic accident more than 1 year ago, after which he had developed ptosis of his left upper eyelid, which did not resolve. He had undergone external levator resection of more than 15 mm of the left upper eyelid, which failed to achieve desired results. On examination,the patient's visual acuity was 20/20 in both eyes and he had normal intraocular pressures. There was no afferent pupillary defect, and the ocular motility full in both eyes. The palebral fissures measured 9 mm on the right and 7 mm on the left with marginal reflex distance of 2.5 mm on the right and 0 mm on the left side with high lid crease [Figure 1a]. Right upper eyelid levator function was greater than 15 mm, and on the left side it was 8-9 mm. Because of patient's acquired ptosis due to trauma and as the subsequent attempt to repair ptosis through external levator resection had failed, it was decided to use a modified Fasanella-Servat procedure under local anesthesia to obtain the desired results [Figure 1b and 1c]. Postoperatively, patient was found to have achieved the desired cosmetic results without complaints of dry eye [Figure 1d].

## DISCUSSION

The Fasanella-Servat procedure for ptosis is of particular interest for residents that are eager to learn the art of ptosis surgery. The procedure has been popular in the past because it corrected minimal ptosis reasonably well and is easy for most general ophthalmologists to perform.^1^ A literature review of the Fasanella-Servat operation revealed that over 30 modifications have been proposed by various ophthalmic surgeons to give them the best results.^2^ Nevertheless, the procedure is useful for patients with minimal ptosis (1–3 mm) and good levator function (9-13 mm), such as those found in some cases of congenital ptosis; residual acquired ptosis, like the one in this report; acquired ptosis associated with Horner's syndrome, senility, myasthenia gravis; post-enucleation ptosis; or ptosis after cataract extraction surgery.^3^ In most cases, the operation may be carried out under local anesthesia with minimal instrumentation and manipulation.

In the basic Fasanella-Servat procedure, the eyelid is gently everted over a Desmarres retractor, and local anesthesia consisting of 2% xylocaine with 1:100,000 epinephrine is infiltrated subconjunctivally above the superior border of the tarsal plate. Two fine curved hemostats (artery forceps) are used to clamp the upper border of the tarsus and lower part of Muller's muscle and conjunctiva. The points of clamps should meet at a level that is approximately above the pupil or at the position where the highest point of the eyelid arch is desired. If a minor adjustment is desired, the amount of trapped tissue can be carefully modified using toothed forceps. Skin and orbicularis muscle should be free of the clamps. Hemostats are placed such that excessive excision of the tissue from the center of the lid is prevented. This avoids peaking of the eyelid arch and creates a more natural appearance.^4^ A double-armed 6-0 plain suture is placed in the medial lid just below the hemostats, and one arm is run laterally through the tissues to be plicated, approximately 1.5 mm beneath the clamps. The clamps are then removed and the tissues within the bed of the crush marks left by the clamps excised, sparing the suture. The other arm of the suture closes the wound edges, and both arms meet at the lateral edge of the eyelid, where the knot can be buried.

The most common complication of tarsomyectomy relates to faulty placement of the clamps. If the clamps are not placed properly, the lid arch may be peaked postoperatively. However, if that happens, 3 to 4 days post-operatively, the suture may be snipped at the site of peaked lid and the lid gently massaged to smoothen the irregularity. Postoperative keratitis is a disturbing problem but is usually self-limited. The use of 6-0 plain suture minimizes suture irritation. Suture granuloma formation is rare, as is postoperative hemorrhage.^5^

### Variations of the Fasanella-Servat Procedure

The multitude of variations of the Fasanella-Servat procedure is not derived because of the lack of its success when used appropriately; the modifications increase its safety or allow use of a relatively simple technique, while enhancing its ability to raise the eyelid. Putterman's Muller's muscle–conjunctival resection is useful for patients with good levator function that have a positive–phenylephrine test (ptosis resolving following instillation of 2.5% phenylephrine in the conjunctival sac).^6^ Good results have been obtained for correcting the ptosis seen in patients with Horner's syndrome. This procedure is similar to Fasanella-Servat tarsomyectomy except that it spares the tarsus. This technique is not effective for patients that have a negative response to phenylephrine test. Gavaris' tarso-Mullerectomy is a modification of the Fasanella-Servat operation; it has the advantage of a more graded result.^7^ It inolves a tarsectomy and shortening of Muller's muscle and conjunctiva. The amount of muscle to be excised depends on the degree of ptosis and the amount of levator function. With mild ptosis and good levator function, 3 mm of tarsus and 4–5 mm of Muller's muscle are resected. In this modified procedure, the eyelid is everted and a horizontal incision is made through the tarsus, 5 to 6 mm from the lid margin. Vertical incisions are made at each end of the tarsal incision, extending upwards 6 to 7 mm. Muller's muscle is dissected free from the levator muscle. Hemostasis is achieved by wet field cautery. Three 6-0 vicryl double-armed sutures are passed through Muller's muscle from the deep surface and they exit on the conjunctival surface. The desired amount of Muller's muscle is excised. The sutures are passed through the edge of the lid fold. The three double-armed sutures are tied directly over the skin. This is an excellent procedure, as it can provide more lid elevation, if desired, compared to the basic Fasanella-Servat operation. It also has the advantage that if at the end of the procedure, a good contour to the eyelid is not achieved, another suture may be added to raise the lid to the desired position.

In the past, corneal irritation from exposed conjunctival sutures was a problem, but the use of softer sutures has eliminated this complication. The entire plicating suture can be buried by passing it at an angle of 45° so that each exit site is the next entrance site. Callahan^8^ suggests wound closure in a step-by-step fashion as the tissue is excised. Closure with a single locked, running 5-0 plain gut suture can be satisfactory after resecting 3 mm of tissue from behind the clamps. Even if no suture is placed, the levator muscle acts to bring the wound margins together to shorten the posterior lamella. Since this operation should only be done if good levator function is present, the sutureless Fasanella-Servat procedure may well succeed. Indeed, Lauring^9^ places the hemostat and crushes and excises the same tissue as in the Fasanella-Servat operation, but he uses no sutures to close the wound.

The modifications of the Fasanella-Servat procedure share certain disadvantages with the original version.^10^ The basic lacrimal secretors are excised and can cause or aggravate keratitis sicca. The loss of partial conjunctiva from the superior formix can lead to contracture and form subsequent cicatricial entropion.^11^ Therefore, tarsomyectomy should be avoided in patients with cicatricial conjunctival disease, such as pemphigoid, conjunctival scarring, amyloidosis, lymphoma, trachoma and conjunctival granuloma. Keratitis sicca may be a relative contraindication since normal conjunctival tissue, including accessory aqueous glands, is excised. More than 3 mm of tarsus should not be excised in the tarsomyectomy procedure. Excision of a larger amount may lead to eyelid instability or entropion and may make later ptosis revision very difficult. Nevertheless, in carefully selected patients with minimal ptosis and good levator function and in whom there are no contraindications, Fasanella-Servat procedure with certain modifications has stood the test of time.
